# Taking Advantage of Nature’s Gift: Can Endogenous Neural Stem Cells Improve Myelin Regeneration?

**DOI:** 10.3390/ijms17111895

**Published:** 2016-11-14

**Authors:** Rainer Akkermann, Janusz Joachim Jadasz, Kasum Azim, Patrick Küry

**Affiliations:** 1 Department of Neurology, Medical Faculty, Heinrich-Heine-University, 40225 Düsseldorf, Germany; Rainer.Akkermann@med.uni-duesseldorf.de (R.A.); janusz.jadasz@uni-duesseldorf.de (J.J.J.); 2 Focus Translational Neuroscience, Institute of Physiological Chemistry, University of Mainz, 55122 Mainz, Germany; kasumazim@googlemail.com

**Keywords:** multiple sclerosis, remyelination, differentiation, cell fate determination, adult neural stem cells, precursor cells, oligodendrocytes, glia, white matter

## Abstract

Irreversible functional deficits in multiple sclerosis (MS) are directly correlated to axonal damage and loss. Neurodegeneration results from immune-mediated destruction of myelin sheaths and subsequent axonal demyelination. Importantly, oligodendrocytes, the myelinating glial cells of the central nervous system, can be replaced to some extent to generate new myelin sheaths. This endogenous regeneration capacity has so far mainly been attributed to the activation and recruitment of resident oligodendroglial precursor cells. As this self-repair process is limited and increasingly fails while MS progresses, much interest has evolved regarding the development of remyelination-promoting strategies and the presence of alternative cell types, which can also contribute to the restoration of myelin sheaths. The adult brain comprises at least two neurogenic niches harboring life-long adult neural stem cells (NSCs). An increasing number of investigations are beginning to shed light on these cells under pathological conditions and revealed a significant potential of NSCs to contribute to myelin repair activities. In this review, these emerging investigations are discussed with respect to the importance of stimulating endogenous repair mechanisms from germinal sources. Moreover, we present key findings of NSC-derived oligodendroglial progeny, including a comprehensive overview of factors and mechanisms involved in this process.

## 1. Introduction

Multiple sclerosis (MS) is an autoimmune disease of the central nervous system (CNS) characterized by the loss of myelin, a specialized membrane produced by oligodendrocytes (OLs) that is essential for normal CNS function. Apart from electrical insulation, facilitating saltatory signal conduction, OLs also provide axons with metabolic and trophic support, such as lactate/pyruvate and neurotrophic factors such as brain-derived neurotrophic factor (BDNF), via the myelin membrane [1,2]. Although the etiology of MS remains unknown, a large body of evidence suggests that activated immune cells target myelinated axons and OLs, leading to OL death and demyelination [3]. While the insult appears to be transient initially with remission following relapses, the occurring damage is progressive. Thus, demyelinated axons are more susceptible, for instance due to lack of physical protection against inflammatory molecules and lack of metabolic and/or trophic support, resulting in neurodegeneration in the long-term [4]. Therefore, disability in MS patients correlates with white matter lesion volume at early stages of disease, whereas disease progression and increased disability is marked by gray matter atrophy [5].

The human CNS has the endogenous potential to repair demyelinated lesions, which so far has been considered to be mediated mainly through the recruitment and differentiation of oligodendrocyte precursor cells (OPCs) [6,7]. These cells are characterized by the expression of certain markers including the basic helix-loop-helix transcription factor Olig2 [8,9], neural/glial antigen 2 (NG2) and platelet-derived growth factor receptor α (PDGFRα) [10,11] and are dispersed throughout the brain parenchyma [12]. The earliest cells of this lineage can be detected around Embryonic Day (E) 8.5, when Olig2 expression precedes that of other OPC markers [8,9]. The use of cell fate mapping techniques revealed the emergence of three distinct waves of OPCs, the first originating from Nkx2.1-positive progenitors in the ventral telencephalon, the second from Gsx2-positive cells in the lateral/caudal ganglionic eminences, and the third wave descending from Emx1-positive cortical progenitor cells [13]. However, not all of these populations contribute equally to the eventual myelinating pool of OLs. This was clearly shown by the finding that Nkx2.1 progenitor-derived OPCs are almost entirely eliminated by early adulthood, whereas the Emx1-derived progenitors remain life-long in the adult brain [13]. There is increasing evidence for the importance of myelinating OLs for the proper functioning of the CNS throughout life, particularly with respect to plasticity and learning [14,15], and the pathology of neurodegenerative disorders, the most prevalent one being MS [16].

For remyelination to occur, OPCs need to be activated, recruited to lesion sites and to subsequently differentiate into myelinating OLs [17]. These cells can then remyelinate denuded axons, a process which can be successful in MS patients [6,18,19]. However, this process is prone to reduced efficiency during the course of disease progression [20]. Whilst OPCs can be detected in and around MS lesions, they often fail to differentiate, probably due to the presence of multiple differentiation-associated inhibitors, which prevent the generation of remyelinating OLs [12,21,22,23,24,25,26]. Therefore, it is crucial that in addition to modulating the immune response, as current MS treatments have been designed for, newer strategies are required for promoting repair mechanisms in MS patient’s CNS, overcoming this inhibitory block in order to counteract progressive damage and provide neuroprotection by remyelination of denuded axons.

**Figure 1:**
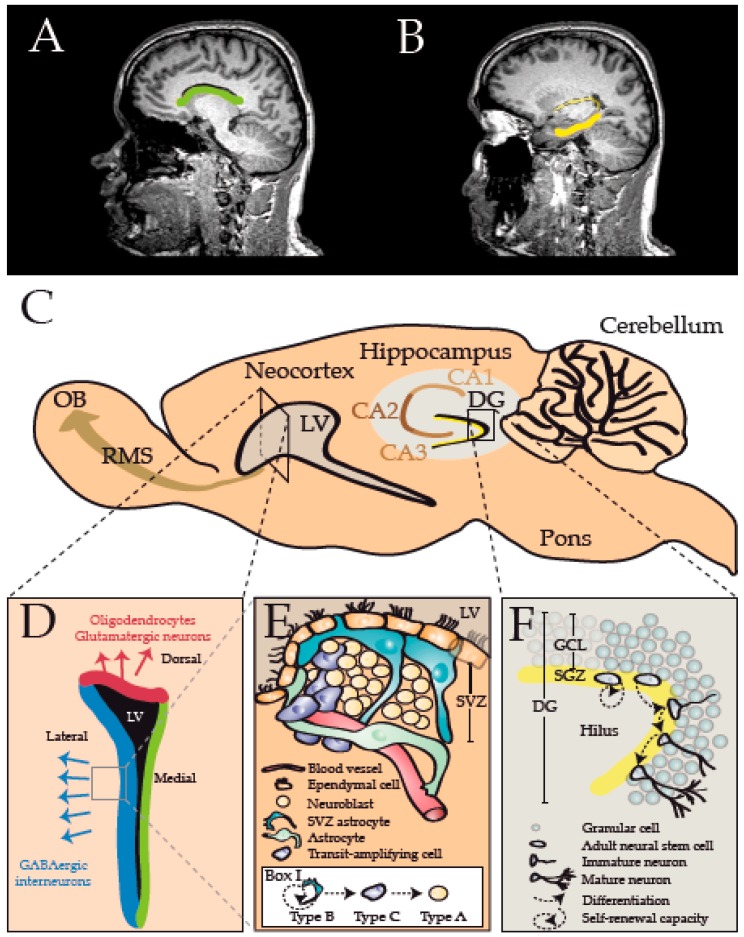
The mammalian central nervous system (CNS) comprises two major stem cell niches: the subventricular zone (SVZ) lining the lateral ventricles (LV), and the subgranular zone (SGZ) in the dentate gyrus (DG) of the hippocampus, as depicted in the human brain (green and yellow areas on magnetic resonance images (MRI) in (**A**) and (**B**), respectively) and rodent brain (**C**); (**D**) The SVZ (coronal view) can be divided into different microdomains based on the progenies of neural stem cells (NSCs): whereas the dorsal domain (red) produces predominantly oligodendrocytes (OLs) and glutamatergic neurons, the lateral domain (blue) generates mainly GABAergic interneurons, and the medial domain is largely non-neurogenic after 2.5 months of life; (**E**) A detailed view of the lateral SVZ shows the microarchitecture of this niche, including self-renewing NSCs (Type B/SVZ astrocyte) that can give rise to transit-amplifying cells (Type C cells). These multipotent progenitors can produce neuroblasts (Type A cells) as well as glial cells, including OLs (Box I). Newly formed neuroblasts usually migrate along the rostral-migratory stream (RMS) to the olfactory bulb (OB), whereas cells determined to become OLs usually migrate into nearby white matter tracts; (**F**) A close-up view of the SGZ depicts how NSCs (Adult neural stem cells) within the hilus of the DG can self-renew or give rise to neurons that are incorporated into the granular cell layer (GCL). In addition to neuronal cells, NSCs within the SGZ also have the potential generate OLs.

A promising strategy has emerged through recent studies, which have identified CNS resident adult neural stem cells (NSCs) as an alternative source of progenitor cells with a high regenerative potential. Even though it has long been debated whether or not the CNS indeed harbors multipotent, self-renewing stem cells capable of replacing cells that may be lost during normal homeostasis or pathological events, it was reported as early as in 1890 that CNS cells actively undergo mitosis [27]. A more detailed analysis by Allen in 1912 already revealed that a thin layer of tissue surrounding the lateral ventricles is one of the most active sites of cell division in the postnatal CNS [28]. At that time he had thus discovered one of the two main germinal niches that persist in the adult mammalian brain, the subventricular zone (SVZ) of the lateral ventricle wall, the second niche being the subgranular zone (SGZ) in the dentate gyrus of the hippocampus (Figure 1) [29,30,31,32,33]. Both regions contain NSCs, which were found to share molecular and morphological hallmarks with astrocytes [34]. These cells express the astroglial markers glial fibrillary acidic protein (GFAP), glutamate-aspartate transporter (GLAST) and brain lipid binding protein (BLBP), and can additionally be identified by nestin and Sox2 expression [34,35,36,37,38]. NSCs are multipotent and their progeny are highly migratory cells that can give rise to neurons, astrocytes and OLs [39]. Notably, these cells were shown to generate myelinating OLs in response to white matter damage, both in animal models [40,41,42,43,44] as well as in humans [45], and were found to extensively contribute to remyelination. Therefore, activation and mobilization of endogenous NSCs presents a promising new strategy in the quest for regenerative, as opposed to immunomodulatory MS therapies.

Several reviews have comprehensively covered OPCs’ capacities for remyelination but lesser attention has been given to their upstream counterparts, the NSCs. In this review, we will discuss the benefits and limitations of endogenous versus exogenous stem cell-mediated therapies for the treatment of demyelinating disorders. This will include an overview of the different subpopulations of NSCs in the CNS, and how they can contribute to remyelination, focusing on the underlying extrinsic and intrinsic factors that may promote this process.

## 2. Endogenous versus Exogenous Stem Cell-Mediated Repair

Overcoming remyelination failure is a primary goal in current MS research and while immunomodulatory drugs have been a major advancement in the treatment of MS patients [46] such drugs to date were not shown to exert concomitant regenerative effects. Remarkably, attempts in supporting regeneration under demyelinating CNS conditions were already made more than 20 years ago. In one of the first studies the authors injected purified oligodendrocyte type-2 astrocyte (O-2A) progenitor cells into demyelinated spinal cord lesions of adult rats and were thus able to remyelinate [47]. Many studies followed achieving functional recovery using different types of cells in different animal models and the results clearly demonstrated that depending on the type and differentiation state of cells as well as the route of administration (intravenously, intraventricularly or intracerebroventricularly), the mechanisms of action and efficiency of repair differed [48]. Thus, injection of undifferentiated NSCs or mesenchymal stem cells (MSCs) generally contributes to repair by either modulating indirectly the central- or peripheral immune environment or by providing trophic support for neuronal or glial cells. On the other hand, in order for transplanted cells to actively contribute to remyelination, oligodendroglial cells were found to require a preconditioning step for programming an OL fate [47,49,50]. Nonetheless, two recent studies showed that NSCs generated either from induced pluripotent stem cells (iPSCs) [51] or from embryonic stem cells [52] that were injected into acutely injured spinal cords of mice efficiently differentiated into OLs and remyelinated axons. Another study showed that injecting intravenously or intracerebroventricularly into experimental autoimmune encephalomyelitis (EAE) mice NSCs derived from the adult SVZ had partly differentiated into OLs, astrocytes or neurons as well as a proportion remaining quiescent [53]. In these mice, transplanted NSCs were also initially found in various other tissues, including lung, liver, spleen and kidney, although they were no longer detected at later time points. Therefore, it remains questionable whether transplantation of multipotent precursor cells is a viable option in promoting CNS repair.

In MS patients, stem cell transplantation has similarly been performed. The two types of transplantation that have been applied so far are autologous hematopoietic stem cell transplantation (AHSCT) and injection of MSCs. In AHSCT, the dysregulated immune system of a patient is eliminated by chemotherapy whereupon previously isolated HSCs from the patient’s bone marrow are transplanted to generate a new immune system. Injection of MSCs, also derived from the patient’s bone marrow, is related to the many beneficial and regenerative effects that could be attributed to these cells [54,55]. While both treatments have yielded rather positive results [56], clinical trials so far have been preliminary and included low patient numbers only. In addition, stem cell transplantation is a complex intervention with an increased risk of complications and serious adverse effects [57], rendering these treatments only reasonable for a small subset of patients suffering from aggressive and fast progressing disease forms.

While the idea of applying stem cell transplantation to treat MS patients has been considered for some time now, mobilizing endogenous NSCs for CNS repair is rather new and unexplored. Based on recent investigations demonstrating the high regenerative potential of this population, this strategy could provide an alternative and potent clinical intervention improving patient’s general conditions, as well as decelerating neurodegeneration and thus reducing the socioeconomic impact of this disease. Ideally, application of therapeutic agents for recruiting NSCs and driving oligodendrogenesis, possibly administered in combination with existing immunomodulatory therapies may be additive, i.e., simultaneously regulating autoimmunity and promoting repair in MS patients. In the following sections, we will briefly describe the origin and characteristics of NSCs as well as discuss the potential and mechanisms of this population in contributing to oligodendrogenesis and subsequent myelin repair.

## 3. Neural Stem Cell Populations of the Central Nervous System

The first type of stem cells of the nervous system are neuroepithelial cells of the developing telencephalon, which self-renew but also produce embryonic radial glial cells [58]. Radial glial cells first appear around E11.5 within the embryonic ventricular zone. They will generate quiescent NSCs (also called type B1 cells) between E13.5 and E15.5, and these cells are activated in postnatal life [59]. In the SVZ following birth, there are quiescent and activated subtypes of NSCs that likely shuttle between these differentiation states [60]. Both subtypes are capable of dividing asymmetrically to self-renew [61]. The activated forms of NSCs differentiate into transit-amplifying progenitors (TAPs/type C cells), which in turn produce neuroblasts (type A cells) that migrate along the rostral migratory stream (RMS) to the olfactory bulb to become inhibitory neurons in most mammals [62,63]. It was recently described that neuroblasts derived from the human SVZ do not migrate as chains into the olfactory bulb, but instead populate the striatum [64,65]. In the second niche, the SGZ, NSCs form new glutamatergic granular neurons that are incorporated into the granular cell layer of the dentate gyrus [66].

The walls of the lateral ventricle have different embryonic origins and, although once thought to be a homogeneous population, newer studies show that NSCs give rise to specific neuronal or glial subtypes depending on their time of generation and site of origin, that is the dorsal and lateral (also ventral) SVZ (Figure 1) [13,67,68,69,70]. Microtransplantation experiments have shown that this fate restriction occurs early in development and is likely to be, at least partly, cell dependent as no re-specification occurs when cells are heterotopically transplanted [71,72,73]. Genome wide transcriptional studies of isolated NSCs from spatially distinct SVZ microdomains of the postnatal and young adult SVZ have been resolved [74,75]. These have identified a surprising degree of transcriptional heterogeneity among subpopulations of NSCs that are largely attributed to the expression of downstream signaling and transcriptional cues as major hallmarks. For example, dorsalization of the SVZ was demonstrated to be regulated at least in part by Wnt/β-catenin signaling [76,77]. The dorsal SVZ is the default source of origin of OL lineage cells compared to the lateral SVZ that generates mainly olfactory interneurons [76,77,78].

The capacity of NSCs to undergo oligodendrogenesis is significantly increased in response to demyelinating insults, at least by SVZ-NSCs [40,41,42,43,45,79,80,81]. However, SGZ-NSCs can also be directed to generate OLs in vitro [54,82,83] and in vivo [84,85,86,87,88]. In order to take advantage of the regenerative potential of NSCs for translational purposes, it is crucial to uncover the underlying changes in the transcriptional pathways that enable NSC-mediated oligodendrogenesis.

## 4. How Do NSCs Respond to Demyelination and Contribute to Myelin Regeneration?

Following the discovery that newly formed parenchymal OPCs (pOPCs) are present in MS brains but increasingly fail to drive myelin regeneration during disease progression [21,22,23,24,25], research in this area has focused on the mechanisms by which remyelination could be enhanced by promoting pOPC-mediated repair [89]. Currently, a number of relevant pathways and factors have been identified and efforts are undertaken in order to translate these findings into pharmacological approaches [90,91]. In recent years, however, growing attention has been paid to the investigation of NSCs as an alternative (or additional) source of regenerative oligodendroglial cells. Several studies have demonstrated that in both EAE as well as toxin- or virus-mediated demyelination SVZ-derived NSCs proliferate and generate progeny that migrate and differentiate into OPCs and mature myelinating OLs which significantly contribute to remyelination [40,41,42,43,44]. While most of these studies have described a differentiation of NSC progeny to OLs via an OPC stage, it remains to be elucidated whether NSCs always undergo the same differentiation program as pOPCs or if they can also produce additional intermediate stages, or even bypass the immature oligodendroglial stages and directly generate mature OLs. Most importantly, as observed in postmortem tissue, NSC recruitment and oligodendrogenesis was also shown to occur in brains of aged MS patients [45]. To what extent SVZ-NSC-derived OLs have the capacity to produce myelin in demyelinated areas has been addressed in elegant studies using fate mapping approaches with Nestin-Cre transgenic mice [42,43,92]. Remarkably, following cuprizone-mediated demyelination, the authors observed a significantly higher number of NSC-derived OLs as compared to pOPC-derived OLs in regions adjacent to the SVZ [42]. In contrast, greater densities of pOPC-derived OLs as compared to NSC-derived OLs were observed in the midline and lateral regions of the corpus callosum, indicating a complementary distribution and contribution to remyelination by both cell populations, SVZ-NSCs as well as pOPCs [42]. In human postmortem MS brains, polysialylated neural cell adhesion molecule (PSA-NCAM)-positive progenitors were mostly found in periventricular lesions, while about half as many were also detected in cortical lesions, and a proportion of these progenitors were found to co-express oligodendroglial markers as well [45]. Importantly, this study demonstrated for the first time a substantial migratory capacity of NSC progeny into demyelinated lesions in the human brain, underlining the potential of these cells for regeneration [45]. Notably, NSC-derived OLs were observed in the corpus callosum, striatum and fimbria fornix in response to demyelination in mice [40,41,93], suggesting that these cells readily migrate to demyelinated lesions. The full scope of progenitor migration from the SVZ to other more distant regions that are myelinated aside from the white matter regions remains still to be determined. Importantly, in mice, activated NSCs that have successfully given rise to progeny that have migrated to demyelinated lesions readily differentiated into mature OLs, and in these areas remyelination was more extensive and the myelin sheaths were thicker (i.e., of normal physiological thickness) as compared to areas populated by pOPC-derived OLs [42,43]. In particular Emx1-expressing dorsal SVZ-NSCs were found to undergo increased proliferation, recruitment and differentiation into OLs as compared with their lateral counterparts in response to experimental demyelination, in both the corpus callosum as well as the spinal cord [92]. Moreover, genetic ablation of Emx1-expressing dorsal NSCs was found to result in significantly reduced OL numbers at lesion sites during remyelination in response to lysolecithin-mediated demyelination of the corpus callosum, thus confirming the importance of this microdomain for myelin repair [92]. Generally, in response to demyelination, NSCs first become activated and generate progenitors that will migrate to the demyelinated lesion in the corpus callosum as early as two weeks into cuprizone challenge. Subsequently, these early progenitor cells undergo oligodendroglial differentiation around week three of cuprizone administration and mature into OLs thereafter [42,43].

Manipulation of endogenous NSCs using pro-oligodendrogenic factors has been addressed in a few recent studies. The oligodendroglial transcription factor Olig2 was found to be crucial in the generation of OLs from SVZ progenitors [94]. Forced overexpression of Olig2 in SVZ-NSCs increases the number of OPCs that subsequently migrate to the corpus callosum, cortex and olfactory bulb and differentiate into OLs, leading to an increase in myelin density in these regions [94,95]. Similarly, SGZ-NSCs that generate almost exclusively projection neurons could be programmed to generate OLs and contribute to remyelination following diphtheria toxin-mediated demyelination in the hippocampus following overexpression of either Ascl1/Mash1 or Olig2 in these cells [84,85]. In addition, inhibition of GSK3β via intraventricular infusion of its specific inhibitors results in increased myelination during development, and promotes remyelination following lysolecithin-mediated demyelination [77,96]. NSC-specific Sirt1 inactivation also leads to improved remyelination following demyelination in the corpus callosum [81]. Together, these studies provide important proof-of-principle for directing endogenous NSCs into fully remyelinating OLs. Soundarapandian and colleagues [97] were able to demonstrate that by overexpression of the transcription factor Zfp488 in SVZ-NSCs, remyelination from NSC-derived OLs is functionally relevant, as these mice showed improved recovery from motor deficits following demyelination.

How does ageing alter the neurogenic niches and NSC-oligodendrogenesis? Changes within the SVZ include the presence of fewer ventricle-contacting SVZ astrocytes, conversion of astrocytes into ependymal cells, as well as a general reduction in TAP/type C cell and neuroblast numbers and cell proliferation [98]. The mechanisms that lead to the age-related decline in OL turnover are considered similar to those that regulate general neurogenesis, such as longer cell cycle length of NSCs and their progeny, loss of growth factors and upregulation of inhibitors [98]. Interestingly, later in life as neurogenesis declines [99,100,101], the rate of oligodendrogenesis remains more constant compared to the generation of neuroblasts in both niches (SVZ as well as SGZ). Whereas some studies reported that stem cell mediated oligodendrogenesis also decreases with age [99], other investigations showed that NSC-mediated oligodendrogenesis remains uncompromised in the aged RMS and olfactory bulb [64,100]. Crawford and colleagues [92] fate-mapped the progeny of dorsal versus ventral SVZ-NSCs in response to focal demyelination in the corpus callosum of aged mice. They reported that whilst dorsal SVZ-NSCs are efficiently recruited to lesions, their differentiation into OLs is hindered, whereas those that arise from ventral NSC sources are recruited more slowly but differentiate rapidly into OLs, as compared to younger animals [92]. Whilst the percentage of differentiated OLs from dorsal NSCs was diminished by approximately a half, the percentage of ventral NSCs differentiating into OLs remained constant in aged animals as compared to their younger counterparts [92]. As these observations stem from naïve animals that did not receive any treatment, they underline the potential and necessity of boosting NSCs through therapeutic manipulation for improved remyelination responses in later adulthood or ageing. Remarkably, in the aged human brain, new OL lineage cells could still be detected in the brain parenchyma and sites close to the lateral wall of the SVZ, indicating the life-long oligodendroglial potential of NSCs [65]. A major finding in the ageing field is that telomerase reactivation in SVZ-NSCs during later adulthood increases both neurogenesis and oligodendrogenesis dramatically [102]. Further studies are needed for describing the age-related changes in oligodendrogenesis from the SVZ in health and disease.

Given the promising potential of NSCs to form myelinating OLs, further work is required for stimulating this population exogenously for participating in repair in demyelinating diseases since the age-related deficits on the pOPC pool dramatically impair their regenerative potential [103].

## 5. How Is NSC-Oligodendrogenesis Regulated?

In order to exploit the potential of endogenous NSCs, it is essential to understand how NSC-oligodendrogenesis is regulated at the molecular level. There is a growing body of literature identifying important positive and negative regulators of oligodendrogenesis from NSCs. Given that both OPCs as well as NSCs can produce myelinating OLs, it is intriguing whether the factors that promote oligodendrogenesis from these two populations are similar. This may have implications in the properties of OPC- versus NSC-derived OLs and their potential to remyelinate, which may indeed be the case [42,43]. In support of this hypothesis, whole transcriptome studies of purified SVZ-NSCs have revealed that the expression of transcriptional machineries regulating oligodendrogenesis as well as neurogenesis are unusually highly abundant compared to their downstream progenitor counterparts [60,74,75]. This suggests that NSCs may be easier to probe for driving particular cell fates compared to pOPC based on these studies. In the following sections, we review extrinsic and intrinsic oligodendrogenic factors regulating either both populations or NSCs only, as well as the impact of inflammatory stimuli on NSC-oligodendrogenesis.

### 5.1. Extrinsic and Intrinsic Factors Regulating NSC-Oligodendrogenesis that Overlap with Parenchymal Oligodendrogenesis

Rivera and colleagues cultured NSCs with mesenchymal stem cell-conditioned medium (MSC-CM) and observed a strong increase in the number of cells committed to an oligodendroglial fate, characterized by an increase in the expression of Olig2, Olig1 and Nkx2.2 transcripts (Table 1 and Table 2) [82]. One candidate identified in a screen for potential extrinsic factors that may have elicited NSC-oligodendrogenesis was ciliary neurotrophic factor (CNTF), even though its effect was much smaller compared to that of MSC-CM, indicating that additional oligodendrogenic factors are produced by MSCs [82]. A later study demonstrated that CNTF can influence the migration of NSC progeny toward the demyelinated corpus callosum via activation of the Janus kinase/signal transducer and activator of transcription 3 (JAK/STAT3) pathway [104]. The transcription factor Ascl1/Mash1 plays a major role in neurogenesis and its deletion leads to a loss of neuronal progenitors [105], but it also significantly reduces postnatal oligodendrogenesis from dorsal SVZ-NSCs [80]. Consistently, its forced overexpression in SGZ-NSCs induces oligodendrogenesis in these cells and enables them to remyelinate a demyelinated lesion [84,85], demonstrating a significant potential of this transcription factor in manipulating endogenous NSCs for repair. Similarly, forced overexpression of Olig2 in SGZ-NSCs induces the generation of remyelination-competent OLs [84,85]. In addition, overexpression of the transcription factor Zfp488 results in the differentiation of NSCs into remyelinating OLs [97]. Bone morphogenic protein (BMP) 4 signaling increases the numbers of GFAP-positive astrocytes from NSCs [106], whereas intraventricular infusion of the BMP4 antagonist Noggin during demyelination significantly enhances the density of mature OLs and remyelinated axons [107]. Intriguingly, infusion of chordin, another BMP antagonist, reprograms neuroblasts destined for the olfactory bulb to an oligodendroglial fate [93]. Epidermal growth factor receptor (EGFR) signaling was found to enhance the numbers and differentiation of NSCs to OLs [108,109]. Similarly, fibroblast growth factor (FGF) 2, when administered into the lateral ventricle, induces both NSC proliferation as well as an increased differentiation into oligodendroglial lineage cells [110]. Interestingly, both stress (in the form of immobilization) as well as injections of corticosterone induced a shift from neurogenesis to increased oligodendrogenesis in the SGZ of adult rats [88]. Deletion of neurofibromin 1 (Nf1), a gene mutated in neurofibromatosis type 1, was found to direct ectopic oligodendrogenesis from SGZ-NSCs [86]. Stimulation of Wnt/β-catenin signaling via either inhibition of glycogen synthase kinase 3β (GSK3β), infusion of Wnt3a into the lateral ventricles, or virus-mediated overexpression of Wnt3 induces oligodendrogenesis specific to the dorsal SVZ in both development and adulthood [76,77,78]. Notably, GSK3β is a potent negative regulator of the entire OL lineage, i.e., from NSCs to myelinating OLs and inhibition of its activity enhances remyelination [77,96]. Interestingly, inhibition of the cyclin-dependent kinase inhibitor p57kip2 also enhances NSC-oligodendrogenesis [54,111]. When p57kip2 is suppressed in SGZ-NSCs and then transplanted into the intact dorsal rat spinal cord, a significant proportion of these cells displayed higher expression of OL markers [54], suggesting programming OL fate by repressing single genes. All of the above-described factors are particularly interesting as they could be used to manipulate both pOPCs as well as NSCs to improve myelin repair.

### 5.2. Extrinsic and Intrinsic Factors Regulating NSC-Oligodendrogenesis

In addition to the factors described above, acting on both OPCs and NSCs, there is a growing list of factors not expressed by pOPCs, exclusively driving oligodendrogenesis from NSCs (Table 1 and Table 2). One of the downstream mediators of Shh signaling, Gli1, was recently found to exert a Shh-independent inhibitory effect on NSC fate decisions. Both genetic and pharmacological inhibition of Gli1 significantly promoted NSC-recruitment and their differentiation into OLs during remyelination whereas inhibition of canonical Shh signaling proved to be ineffective [114]. Similar to Gli1, inactivation of Sirt1, a protein deacetylase implicated in energy metabolism, enhances oligodendrogenesis from NSCs [81]. Increased NSC-derived OL densities following Sirt1 inactivation correlate with an upregulation of PDGFRα expression and increased levels of phospho-p38 MAP kinase and phospho-AKT, downstream targets of PDGFRα signaling [81]. In addition, NSC-specific Sirt1 inactivation leads to enhanced remyelination following lysolecithin-induced demyelination in the corpus callosum [81]. Whereas overexpression of the transcription factor Nuclear factor I X (NFIX) in NSCs inhibits oligodendrogenesis in vitro, loss of NFIX significantly increases the number of OLs derived from SVZ-NSCs both in vitro and in vivo [115]. Similarly, pre B-cell leukemia homeodomain 1 (Pbx1; a transcription factor) is a positive regulator of neurogenesis that is expressed in rapidly amplifying progenitors and its deletion directs their fate toward oligodendrogenesis from otherwise neurogenesis [116]. Another transcription factor that drives NSC-oligodendrogenesis in the SVZ is prospero-related homeobox 1 gene (Prox1) [117]. Interestingly, Prox1 expression was previously associated with promoting neurogenesis as opposed to oligodendrogenesis in the SGZ [118], clearly demonstrating differences in the microenvironment and factors regulating the two different forebrain niches. Additionally, Prox1 is regarded as a target of Wnt signaling and consistent with previous studies showing that Wnt/β-catenin signaling is primarily detectable in the dorsal SVZ [76,77,78], Prox1 is also predominantly expressed within this microdomain [117]. Finally Drosha, part of the microRNA-processing machinery, stabilizes neurogenesis and its deletion enabled ectopic oligodendrogenesis via recruitment of nuclear factor IB (NFIB) that initiates pro-oligodendroglial transcriptional programs in SGZ-NSCs [87].

It therefore appears that some aspects of the transcriptional coding required for driving oligodendrogenesis from NSCs have been characterized. Further research is now required to better understand how NSCs can be efficiently activated by stimulating key pathways necessary for activation, migration, oligodendroglial differentiation and subsequent remyelination.

### 5.3. Inflammatory Factors Regulating NSC-Oligodendrogenesis

Considering the fact that MS is an autoimmune disease it is imperative to describe how inflammatory molecules, secreted by innate or adaptive immune cells, affect NSC-oligodendrogenesis and their potential for CNS remyelination. Pluchino et al. [121] have shown that persistent inflammation as observed during EAE leads to an inhibition in SVZ-NSC proliferation, as well as a decrease in the generation of PDGFRα-positive cells in this niche. It was also reported that small amounts of ectopically expressed cytokine interferon-γ (IFNγ) enhance neurogenesis whilst diminishing oligodendrogenesis in the SGZ of adult mice [122]. In a different study, the authors injected tumor necrosis factor α (TNFα) and IFNγ into the corpus callosum of EAE mice and detected augmented proliferation in the SVZ and increased oligodendrogenesis at the expense of neurogenesis [123]. Nitric oxide (NO), which is produced by activated microglia and macrophages, was also found to inhibit neurogenesis and to promote oligodendrogenesis from NSCs, via induction of the transcription factor neuron-restrictive silencing factor-1(NRSF)/repressor element-1 silencing transcription (REST) in vitro [124,125]. In addition, H_2_O_2_, another product associated with oxidative stress, increases NSC-derived oligodendrogenesis, and this is correlated with upregulation of Sirt2 expression [126]. During the chronic phase of EAE, inhibition of microglial/macrophage activation using minocycline decreased pathology concomitant with an increased NSC proliferation and their differentiation into OLs [127]. These studies indicate that inflammatory molecules may have different effects on NSC-oligodendrogenesis and remyelination, depending on microenvironment, lesion context as well as specific immune activities and the timely regulation of these. Importantly, a study by Nait-Oumesmar and colleagues [45] demonstrated that despite the obvious prolonged exposure to inflammatory stimuli, the proliferative potential of SVZ-NSCs remained intact in MS brains, even from MS patients aged 80 years. It can therefore be assumed that repetitive inflammatory exposure does not necessarily hinder the capacity to generate new NSC-derived OLs in active and chronic active lesions in MS brains.

## 6. Conclusions

Even though a cure for MS is still elusive, research has made substantial progress in treating affected individuals. Application of immunomodulatory therapies can have profound beneficial effects by reducing relapse rates. The next major step that needs to be undertaken is to successfully target the second major component of the disease: the nervous system. By modulating the immune response and simultaneously supporting and improving endogenous repair processes, we may efficiently break the vicious cycle resulting in progressive neurodegeneration. Whereas stem cell transplantation can be a potent means to treat a subset of MS patients, the majority of patients may require a less invasive and more straightforward therapy. One promising approach is supporting the endogenous sources of stem- and precursor cells that can replace lost OLs and thus restore myelin in demyelinated lesions. As presented in this review, a number of factors have been revealed that can drive oligodendrogenesis from NSCs, and possibly from OPCs concomitantly. Further research is required to substantiate these findings and to identify the most promising candidates to be targeted for biomedical translation.

## Figures and Tables

**Table 1:** Summary of intrinsic factors and their oligodendrogenic effects on NSCs and pOPCs.

Factor	Effect on NSCs	Same Effect on pOPCs?	References
Oligodendrocyte transcription factor 2 (Olig2)	↑ oligodendrogenesis; increased myelination and remyelination	yes	[84,85,94,95]
Nk2 homeobox 2 (Nkx2.2)	↑ oligodendrogenesis; increased myelination and remyelination	yes	[112]
SRY-Box 10 (Sox10)	↑ oligodendrogenesis and remyelination	yes	[84,85]
Achaete-scute homolog 1 (Ascl1)/Mash1	↑ oligodendrogenesis and remyelination	yes	[80,84,85,113]
Zinc finger protein 488 (Zfp488)	↑ oligodendrogenesis	yes	[97]
p57kip2 (Cdkn1c) (*inhibitory*)	↑ oligodendrogenesis upon p57kip2 suppression	yes	[54,111]
Gli1 (*inhibitory*)	↑ recruitment and oligodendrogenesis upon Gli1 inhibition	no	[114]
Sirtuin 1 (Sirt1) (*inhibitory*)	↑ oligodendrogenesis and remyelination upon Sirt1 inactivation	no	[81]
Nuclear factor I X (NFIX) (*inhibitory*)	↑ oligodendrogenesis upon NFIX deletion	no	[115]
B-cell leukemia homeodomain 1 (Pbx1) (*inhibitory*; SVZ specific)	↑ oligodendrogenesis upon Pbx1 deletion	no	[116]
Prospero-related homeobox 1 gene (Prox1)	↑ oligodendrogenesis in SVZ (but ↓ oligodendrogenesis in SGZ)	no	[117,118]
Drosha (*inhibitory*)/ Nuclear factor IB (NFIB)	↑ oligodendrogenesis upon Drosha deletion, via relieve of NFIB repression	no	[87]
Neurofibromin 1 (Nf1) (*inhibitory*)	↑ oligodendrogenesis upon Nf1 deletion	to some extent	[86,119]

**Table 2:** Summary of extrinsic factors and their oligodendrogenic effects on NSCs and pOPCs.

Factor	Effect on NSCs	Same Effect on pOPCs?	References
Ciliary neurotrophic factor (CNTF)	↑ migration toward demyelinated lesions	yes	[104]
Epidermal growth factor receptor (EGFR)	↑ oligodendrogenesis, migration toward demyelinated lesions and increased remyelination	yes	[108,109]
Fibroblast growth factor receptor (FGFR)	↑ oligodendrogenesis (differential expression of FGFR1/2 and FGFR3 in dorsal and lateral SVZ, respectively)	yes	[110]
Wnt/β-catenin	↑ oligodendrogenesis in dorsal SVZ	yes	[76,77,78]
Bone morphogenic proteins (BMPs) (*inhibitory*)	↑ oligodendrogenesis and remyelination upon BMP inhibition	yes	[93,107]
Mesenchymal stem cell (MSC) conditioned medium	↑ oligodendrogenesis	yes	[82,120]
Corticosterone	↑ oligodendrogenesis	yes	[88]
